# Lamin B1 and nuclear morphology in peripheral cells as new potential biomarkers to follow treatment response in Huntington's disease

**DOI:** 10.1002/ctm2.1154

**Published:** 2023-02-13

**Authors:** Marta Garcia‐Forn, Carla Castany‐Pladevall, Arantxa Golbano, Jesús Pérez‐Pérez, Verónica Brito, Jaime Kulisevsky, Esther Pérez‐Navarro

**Affiliations:** ^1^ Departament de Biomedicina, Facultat de Medicina i Ciències de la Salut, Institut de Neurociències Universitat de Barcelona Barcelona Catalonia Spain; ^2^ Institut d'Investigacions Biomèdiques August Pi i Sunyer (IDIBAPS) Barcelona Catalonia Spain; ^3^ Centro de Investigación Biomédica en Red sobre Enfermedades Neurodegenerativas (CIBERNED) Madrid Spain; ^4^ Movement Disorders Unit, Neurology Department Hospital de la Santa Creu i Sant Pau Barcelona Spain; ^5^ Biomedical Research Institute (IIB‐Sant Pau) Barcelona Spain; ^6^ Seaver Autism Center for Research and Treatment Icahn School of Medicine at Mount Sinai New York NY USA

Dear Editor

In neurodegenerative diseases, neuronal dysfunction and degeneration start many years before the emergence of clinical symptoms. An important goal to advance in their knowledge and treatment is the identification of peripherical biomarkers to predict the onset and progression and to test the efficacy of therapies.^1^ We have previously shown that alterations in lamin B1, a member of the lamin family of proteins that are crucial for nuclear functionality,^2^ are involved in the pathophysiology of Huntington's disease (HD). Specifically, lamin B1 levels are increased in the R6/1 HD mouse model at the onset of motor symptoms in striatal medium‐sized spiny and CA1 hippocampal neurons nuclei in correlation with nuclear dysfunction.^3^ Therefore, we asked if increased lamin B1 levels and/or alterations in nuclear morphology could be also occurring in more accessible cells, namely fibroblasts and blood cells, and serve as biomarkers of the disease progression and/or treatment efficacy.

Here, we show that HD patients’ peripheral cells display increased lamin B1 levels and alterations in nuclear morphology in a CAG‐dependent manner as early as at pre‐symptomatic stages. In R6/1 mice, fibroblasts and peripheral blood mononuclear cells (PBMCs) show intact lamin B1 levels but altered nuclear morphology at different stages of the disease, changes that were prevented by betulinic acid (BA) administration. Our data suggest the analysis of lamin B1 protein levels and nuclear morphology in peripheral cells as possible biomarkers for monitoring pharmacological treatments in HD.

Lamin B1 protein levels were analysed by Western blot in primary fibroblast cultures derived from non‐affected individuals (control) and from HD patients (Table S1). HD patients’ fibroblasts displayed increased lamin B1 levels in comparison to control ones (Figure 1A) with no correlation with the stage of disease progression, the presence or not of depression, or age (Figure S1A–C). Hence, HD fibroblasts were classified depending on the number of CAG repeats. The CAG‐repeats threshold at 42 was stabilised by paring CAG repeat length (*Y* axis) with lamin B1 protein levels (*X* axis) for each individual in a scatter plot (Figure 1B). A positive correlation between lamin B1 levels and the number of CAG repeats was observed (Figure 1C). Accordingly, only fibroblasts expressing mHTT with ≥42 CAG repeats showed increased lamin B1 levels (Figure 1D), which was detected from pre‐symptomatic stages (Figure 1E) in all patients independently of the presence of depression (Figure 1F), an early HD symptom.^4^ Moreover, fibroblasts from HD patients expressing mHTT with ≥42 CAG repeats showed an increase in the presence of nuclear blebs, protrusions that correlate with nuclear functional alterations^5^, ^6^ (Figure 1G). Lamin B1 levels tended to be increased in those nuclei showing blebs in both control and HD fibroblasts (Figure S1D), a sign of correlation between elevated lamin B1 levels and the presence of blebs. BA administration to R6/1 mice reduces lamin B1 levels in a brain region‐dependent manner.^3^ In‐line with our previous results, lamin B1 levels were normalized in HD patients’ fibroblasts treated with BA, whereas this treatment had no effect on control ones (Figure S1E,F).

**FIGURE 1:**
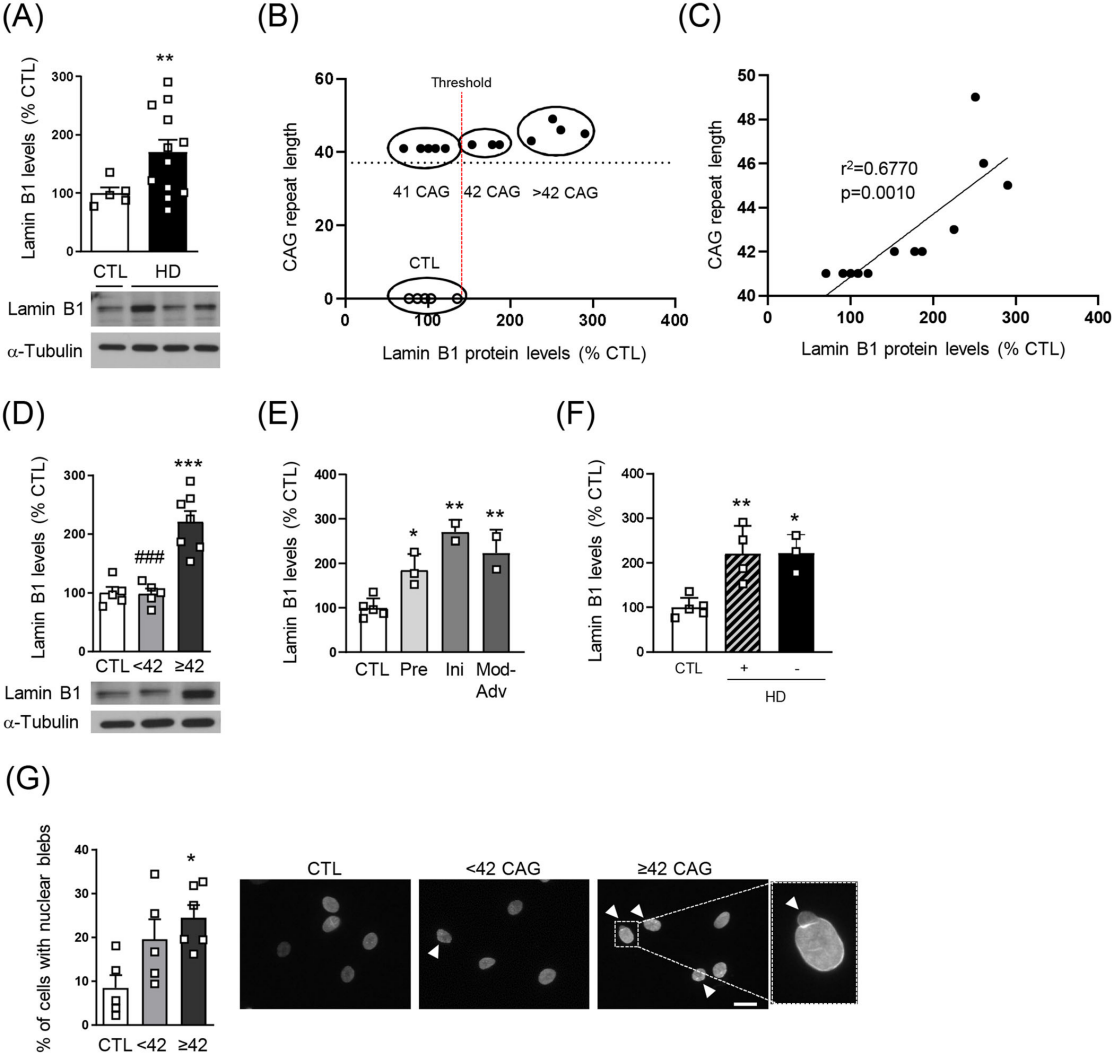
Lamin B1 protein levels and nuclear blebs in fibroblasts from Huntington's disease (HD) patients. (A) Lamin B1 protein levels were analysed by WB in protein extracts from fibroblasts of control (CTL) individuals and HD patients. (B) Scatter plot representing the CAG repeat length (*Y* axis) and fibroblasts’ lamin B1 protein levels (*X* axis) for each individual (CTL: empty circles; HD patients: full circles). The threshold for classifying HD fibroblasts depending on CAG repeat length is 42. (C) Correlation between lamin B1 levels (*X* axis) and CAG repeat number (*Y* axis). (D) Lamin B1 protein levels in fibroblasts of control (CTL) individuals and HD patients expressing mHTT with <42 or ≥42 CAG repeats analysed by WB. (E) Lamin B1 protein levels in HD patients expressing mHTT with ≥42 CAG repeats classified depending on the disease stage (pre: presymptomatic; Ini: initial stage; Mod‐Adv: moderate‐advanced stage). (F) Lamin B1 protein levels in HD patients expressing mHTT with ≥42 CAG repeats classified depending on the presence (+) or not (−) of depression. (G) The percentage of cells presenting nuclear blebs was analysed by immunohistochemistry using anti‐lamin B1 antibody followed by stereological counting in fibroblasts from non‐affected individuals (CTL) and HD patients expressing mHTT with <42 or ≥42 CAG repeats. Representative images are shown. Scale bar 25 μm. (A, D–F) Values (obtained by densitometric analysis of WB data; α‐tubulin used as loading control) are expressed as percentage of controls. Representative immunoblots are shown. (A, D–G) In graphs, each point corresponds to the value from an individual sample. Bars represent the mean ± S.E.M. One‐way ANOVA followed by Bonferroni's post hoc test. *p < .05; **p < .01; ***p < .001 compared to CTL values; ^###^ *p* < .001 compared to ≥42 values

Lamin B1 protein levels were also analysed in PBMCs by FACSI (see Table S2). Only B lymphocytes from HD patients showed altered lamin B1 levels (Figure 2A) with a significant increase in those expressing mHTT with ≥42 CAG repeats (Figure 2B), whereas nuclear circularity was only affected in those with <42 CAG repeats. We did not observe differences in lamin B1 intensity levels between disease stages nor did we observe an effect on lamin B1 levels due to age (Figure S2A,C). Moreover, lamin B1 increased levels were only detected in B lymphocytes from HD patients without depression (Figure S2B).

**FIGURE 2:**
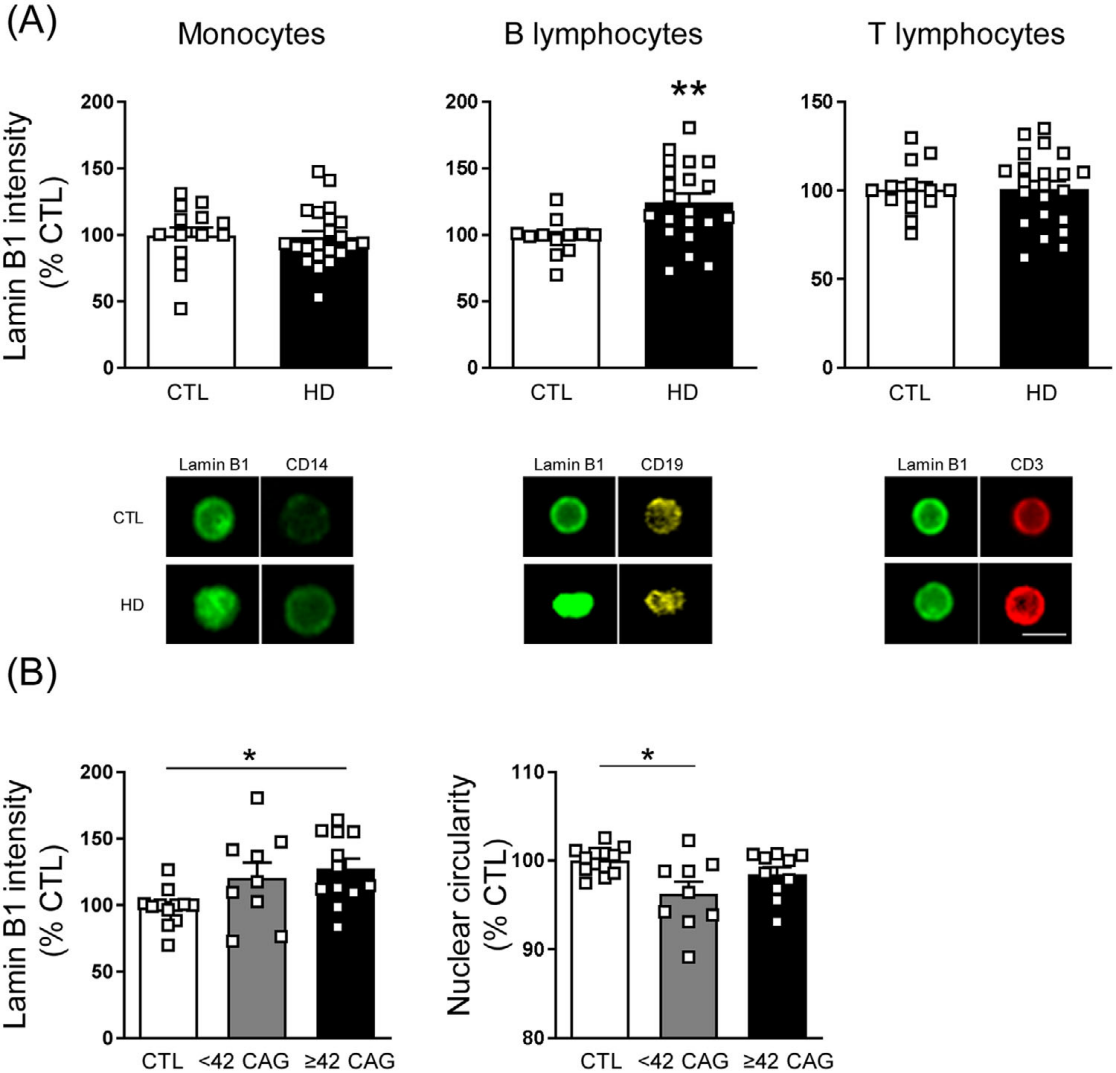
B lymphocytes from Huntington's disease (HD) patients present increased lamin B1 levels and altered nuclear circularity. (A) Lamin B1 intensity levels (as means of integrated densities) were analysed by FACSI in different peripheral blood mononuclear cell (PBMC) types from HD patients and control individuals (CTL). Representative images are shown. Scale bar 10 μm. (B) Nuclear circularity was analysed in B lymphocytes from control individuals (CTL) and from HD patients expressing mHTT with <42 or ≥42 CAG repeats. In graphs, each point corresponds to the value of an individual. Bars represent the mean ± S.E.M. (A) Two‐tailed unpaired Student's *t* test; *p < .05 and (B) one‐way ANOVA followed by Bonferroni's post hoc test; **p < .01

Mouse models allow to obtain longitudinal data that permit to correlate changes in peripheral cells with alterations in brain neurons. Thus, we asked whether fibroblasts and PBMCs from R6/1 mice also show lamin B1 alterations. Moreover, as BA administration normalizes lamin B1 levels in the R6/1 mouse brain and in HD patients’ fibroblasts, we analysed if the effects of this treatment could be monitored peripherally. Wild‐type and R6/1 mice were treated from 8 to 20 weeks of age and blood, and fibroblasts were collected at different time points (Figure 3A). In comparison to vehicle‐treated wild‐type mice, the number of fibroblasts presenting nuclear blebs was increased from 12 to 20 weeks of age in fibroblasts from vehicle‐treated R6/1 mice and that was prevented by BA administration (Figure 3B,C). However, lamin B1 levels were not altered at any of the ages analysed (Figure S3A). Moreover, in 20‐week‐old mice, lamin B1 levels were increased in those fibroblasts showing nuclear blebs (Figure S3B), suggesting that increased lamin B1 levels are involved in the formation of blebs. For the analysis of lamin B1 levels and nuclear morphology in PBMCs, we performed immunohistochemistry in blood films as not enough quantity of blood was obtained for FACSI. PBMCs from 16‐ to 20‐week‐old vehicle‐treated R6/1 mice displayed altered nuclear circularity in comparison to vehicle‐treated wild‐type littermates (Figure 4A), although lamin B1 intensity was not altered at any of the ages analysed (Figure S4A). Interestingly, BA administration delayed the appearance of this phenotype until 20 weeks of age (Figure 4A). Moreover, in PBMCs with altered nuclear circularity at 20 weeks of age, lamin B1 levels tended to be increased (Figure S4B).

**FIGURE 3:**
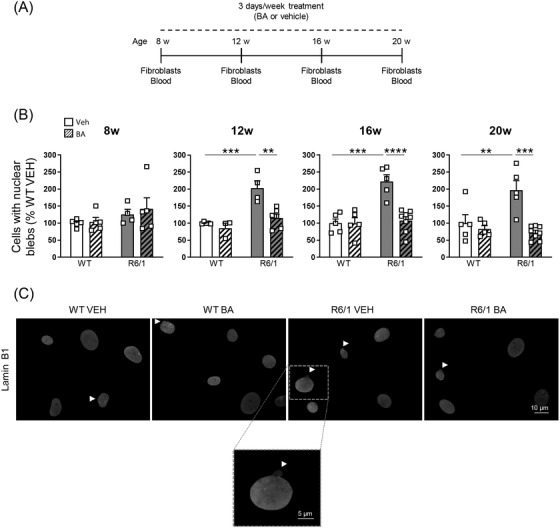
Betulinic acid treatment prevents the appearance of nuclear blebs in fibroblasts from R6/1 mouse. (A) Timeline of the experimental procedure used to assess the peripheral effects of betulinic acid (BA) in wild‐type (WT) and R6/1 mice. Blood and fibroblasts samples from vehicle‐ and BA‐treated WT and R6/1 mice were obtained at the indicated week (w) of treatment. (B) The percentage of cells presenting nuclear blebs was analysed by immunohistochemistry in fibroblasts from vehicle‐ or BA‐treated WT and R6/1 mice at different stages of the disease (w, weeks). Each point corresponds to the value of an individual sample. Bars represent the mean ± S.E.M. (C) Representative images at 16 weeks of age are shown (white arrowheads: blebs). Scale bar 10 μm. One‐way ANOVA followed by Bonferroni's post hoc test. **p < .01, ***p < .001, ****p < .0001

**FIGURE 4:**
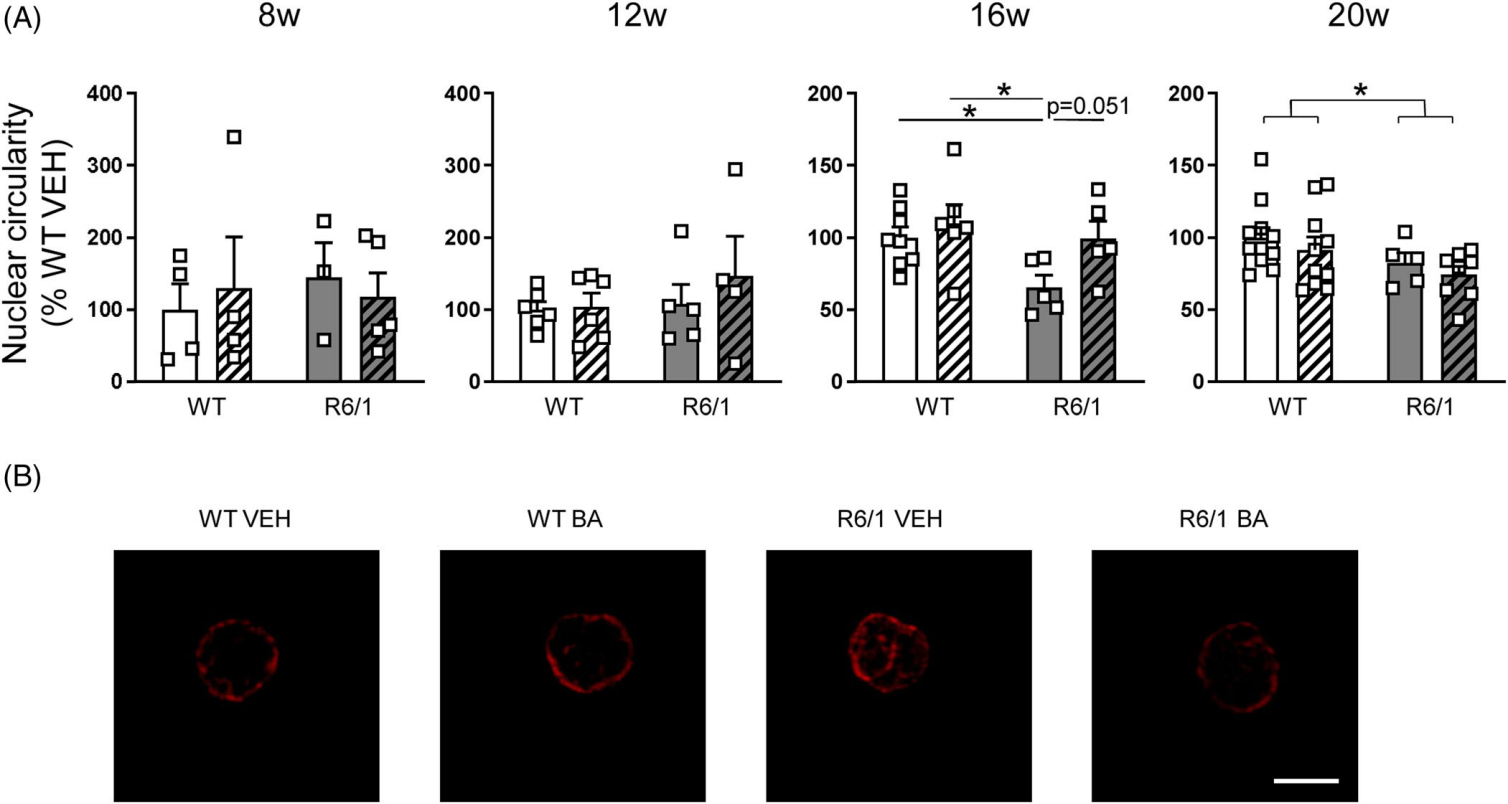
Betulinic acid (BA) treatment delays the appearance of lamin B1 alterations in peripheral blood mononuclear cells (PBMCs) from R6/1 mice. (A) Nuclear morphology was analysed by immunohistochemistry in PBMCs from vehicle‐ and BA‐treated wild‐type (WT) and R6/1 mice at different disease stages of the disease (w, weeks). Each point corresponds to the value from an individual sample. Bars represent the mean ± S.E.M. (B) Representative images at 16 weeks of age are shown. Scale bar 5 μm. One‐way ANOVA followed by Bonferroni's post hoc test. *p < .05

In conclusion, lamin B1 protein levels are increased in HD patients’ fibroblasts and B lymphocytes in a CAG length‐dependent manner, correlating with alterations in nuclear morphology. In the R6/1 mouse model, although lamin B1 levels are not altered, fibroblasts and PBMCs show altered nuclear morphology that is ameliorated by BA administration (Figure S5). Interestingly, the detection of these changes in stages previous to the development of symptoms, both in HD patients and R6/1 mice, could help to an earlier implementation of neuroprotective therapies. Overall, our results support the usefulness of lamin B1 and nuclear morphology analysis in HD patients’ fibroblasts or B lymphocytes to peripherally monitoring the effectiveness of drug treatments.

## CONFLICTS OF INTEREST

The authors declare no conflicts of interest.

## Supporting information

Supporting Information

Supporting Information

Supporting Information

Supporting Information

Supporting Information

Supporting Information

Supporting Information
