# Supplementary figures and images for: Lamin B1 and nuclear morphology in peripheral cells as new potential biomarkers to follow treatment response in Huntington's disease
Source: Clin Transl Med. 2023 Feb 13;13(2):e1154. doi: 10.1002/ctm2.1154 (PMC9925371; doi:10.1002/ctm2.1154)

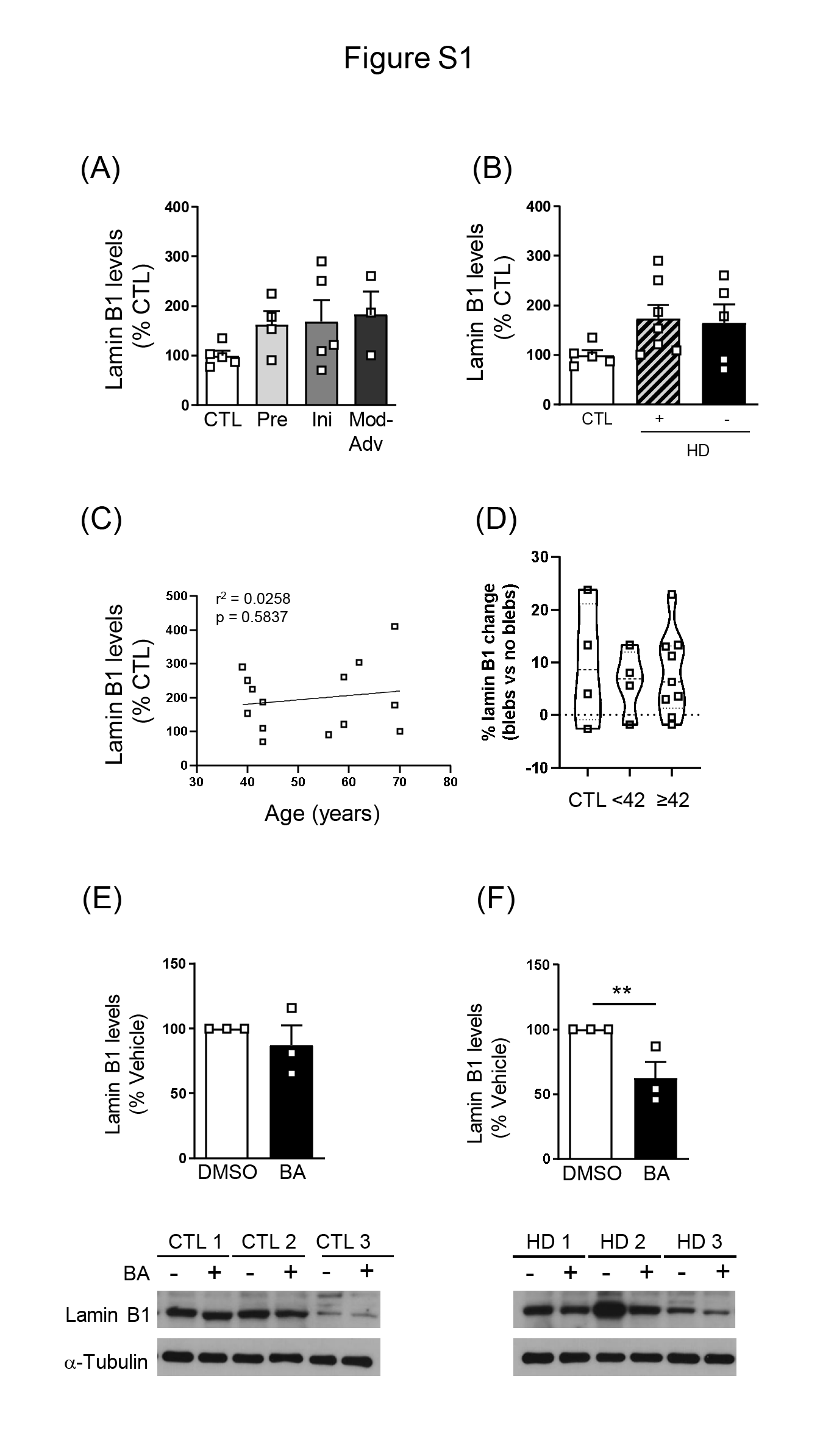

Supplement: Supplementary file 1 — Supporting Information [file CTM2-13-e1154-s002.tif]

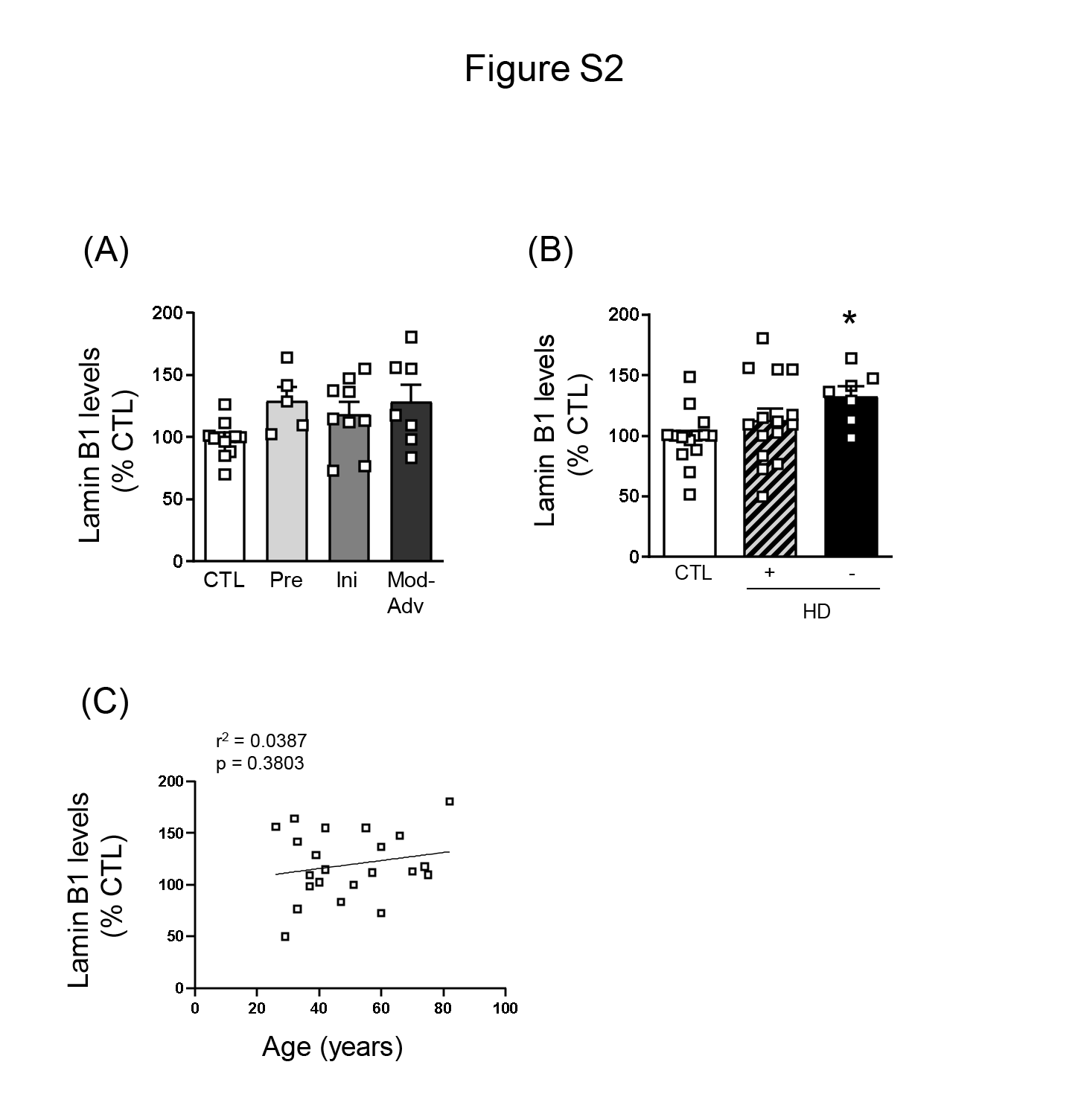

Supplement: Supplementary file 2 — Supporting Information [file CTM2-13-e1154-s007.tif]

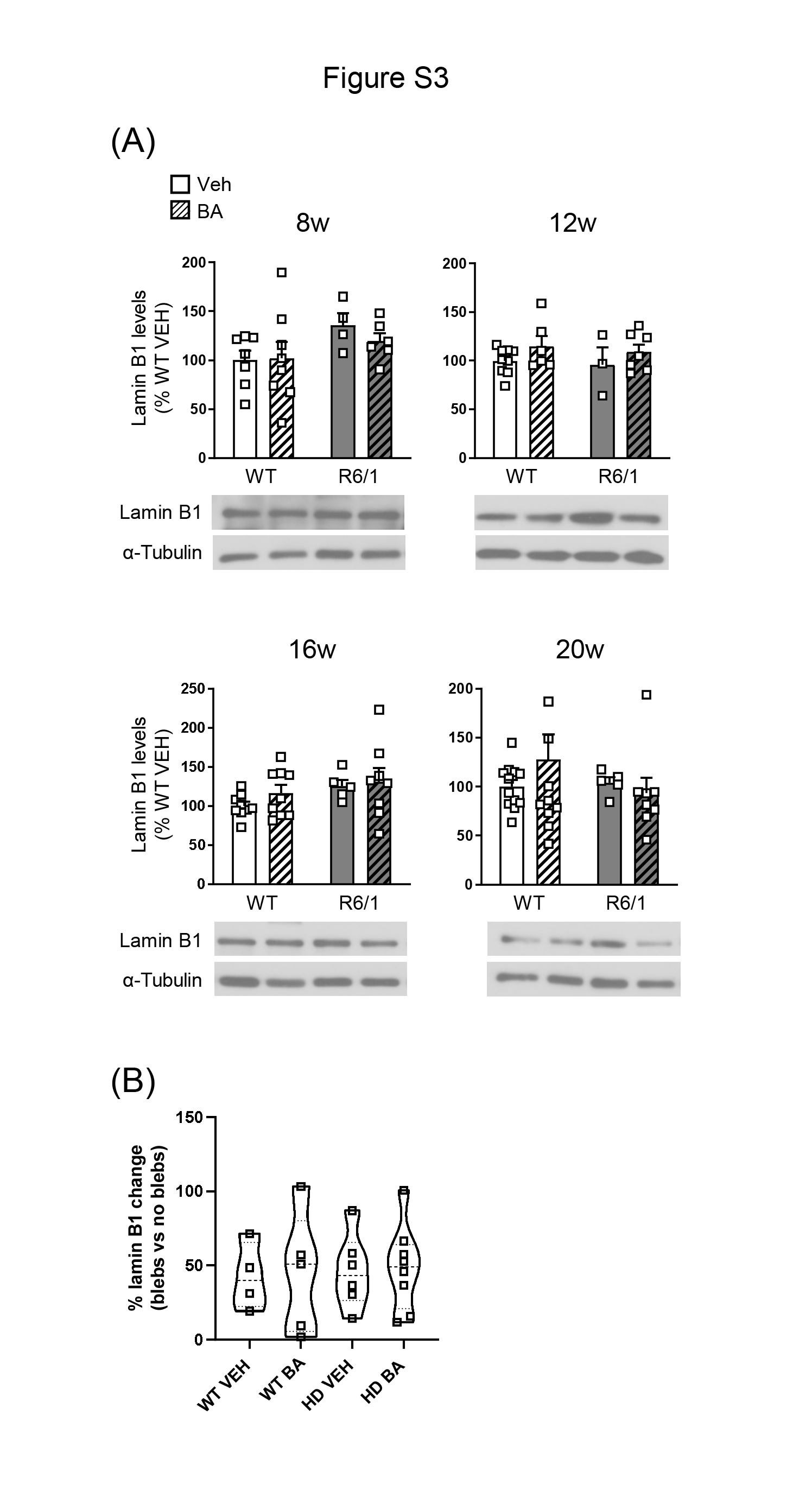

Supplement: Supplementary file 3 — Supporting Information [file CTM2-13-e1154-s005.tif]

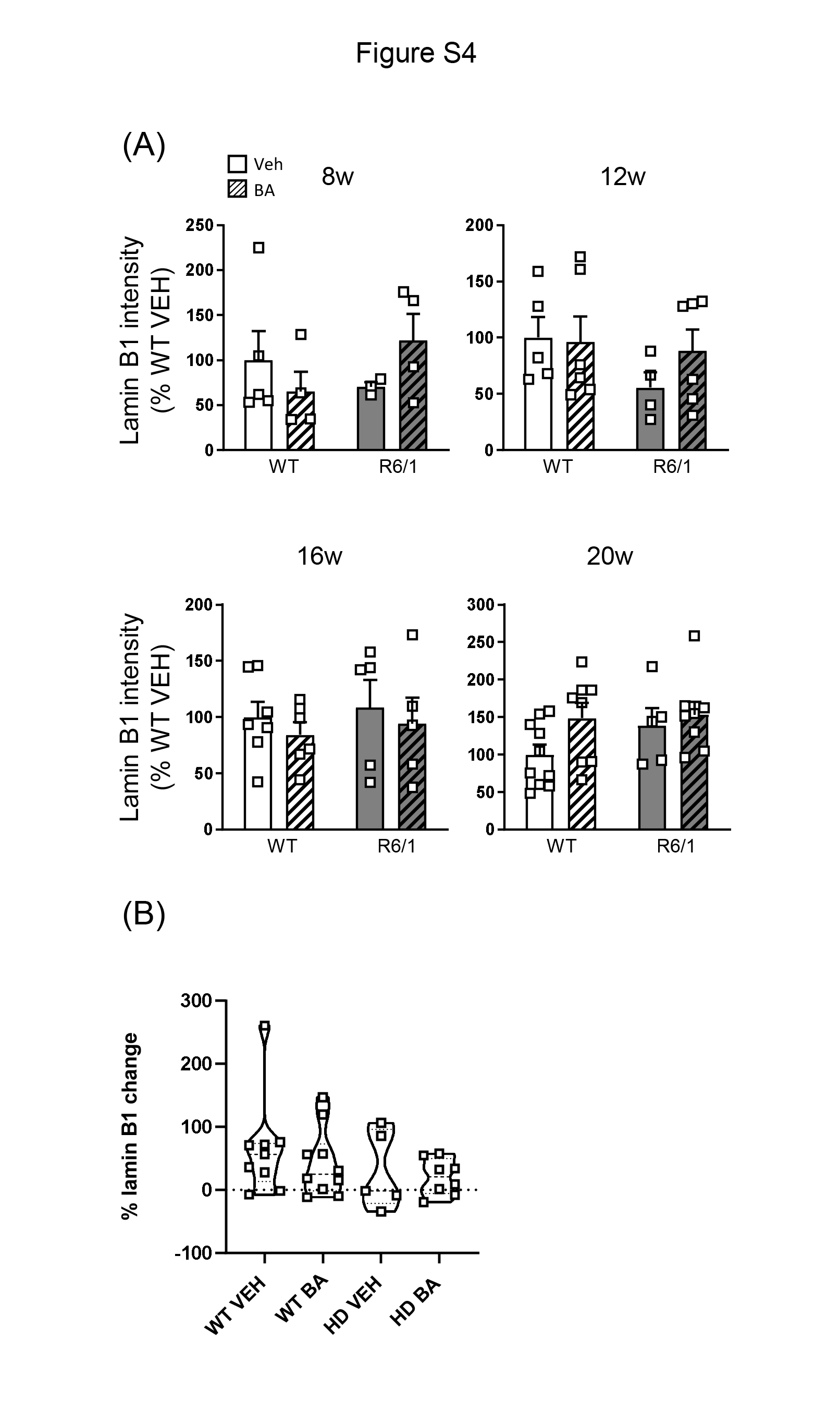

Supplement: Supplementary file 4 — Supporting Information [file CTM2-13-e1154-s004.tif]
