# Supplementary material for: Lamin B1 and nuclear morphology in peripheral cells as new potential biomarkers to follow treatment response in Huntington's disease
Source: Clin Transl Med. 2023 Feb 13;13(2):e1154. doi: 10.1002/ctm2.1154 (PMC9925371; doi:10.1002/ctm2.1154)
Supplement: Supplementary file 5 — Supporting Information [file CTM2-13-e1154-s001.docx]

Figure S1 Analysis of lamin B1 in fibroblasts from HD patients and response to betulinic acid (BA) administration. Lamin B1 protein levels were analyzed by western blot and were classified depending on (A) disease stage (CTL: non-affected individuals; Pre: presymptomatic; Ini: initial stage; Mod-Adv: moderate-advanced stage or (B) presence (+) or not (-) of depression. (C) Graph shows the correlation between lamin B1 levels and age in fibroblasts from HD patients. Values (obtained by densitometric analysis of western blot data; α-tubulin used as loading control) are expressed as percentage of controls (non-affected individuals). (D) Violin plot showing the percentage of lamin B1 increase in fibroblasts showing nuclear blebs in non-affected individuals (CTL) and HD patients expressing mHTT with < or ≥ 42 CAG repeats. CTL, mean: 9.65, SD: 11.46; <42 CAG, mean: 6.3, SD: 6.30; ≥ 42 CAG, mean: 7.91, SD: 7.9. (E-F) Lamin B1 protein levels were analyzed by Western blot in fibroblasts from (E) control individuals (CTL) and (F) HD patients expressing mHTT with ≥ 42 CAG repeats, 48 h after treatment with BA (20 μg/ml) or the vehicle DMSO. Images show immunoblots with all the samples analyzed. In all graphs, each point corresponds to the value from an individual. Bars represent the mean ± S.E.M. (A-B) One-way ANOVA followed by Bonferroni’s post hoc test. **p* <.05. (E-F) Two-tailed unpaired Student’s t test; ***p* < .01.

Figure S2 Lamin B1 levels in B lymphocytes from HD patients. Lamin B1 levels were analyzed by FACSI (as means of integrated densities) and classified depending on (A) disease stage (CTL: non-affected individuals; Pre: presymptomatic; Ini: initial stage; Mod-Adv: moderate-advanced stage or (B) presence (+) or not (-) of depression. Values are expressed as a percentage of controls (non-affected individuals). (C) Graph shows the correlation between lamin B1 levels (as means of integrated densities) and age in B lymphocytes from HD patients. In all graphs, each point corresponds to the value from an individual. Bars represent the mean ± S.E.M. (A-B) One-way ANOVA followed by Bonferroni’s post hoc test. **p* <.05.

Figure S3 Lamin B1 levels are not altered in fibroblasts from R6/1 mice. (A) Lamin B1 protein levels were analyzed by western blot in protein extracts obtained from fibroblasts of vehicle- or BA-treated wild-type (WT) and R6/1 mice at different stages of the disease. Values (obtained by densitometric analysis of western blot data; α-tubulin used as loading control) are expressed as a percentage of vehicle-treated WT mice. Bars represent the mean ± S.E.M. Representative immunoblots are shown. (B) Violin plot showing the percentage of lamin B1 increase (analyzed by immunocytochemistry) in fibroblasts showing nuclear blebs in WT and R6/1 mice at 20 weeks of age. WT VEH, mean: 42.51, SD: 22.62; WT BA, mean: 44.43, SD: 40.89; HD VEH, mean: 46.04, SD: 25.22; HD BA, mean: 48.37, SD: 28.54. In all graphs, each point corresponds to the value from an individual mouse. One-way ANOVA followed by Bonferroni’s *post hoc* test.

Figure S4 Lamin B1 levels are not altered in PBMCs from R6/1 mice. (A) Lamin B1 intensity (analyzed as integrated density by immunohistochemistry) in PBMCs from vehicle- and BA-treated wild-type (WT) and R6/1 mice at different weeks of age. (B) Percentage of lamin B1 increase in PBMCs showing low circularity in 20-week-old mice. WT VEH, mean: 65.58, SD: 79.36; WT BA, mean: 42.43, SD: 53.76; HD VEH, mean: 29.50, SD: 62.17; HD BA, mean: 20.21, SD: 28.76. Each point corresponds to the value from an individual mouse. Bars represent the mean ± S.E.M. One-way ANOVA followed by Bonferroni’s *post hoc* test.
