# Supplementary material for: Lamin B1 and nuclear morphology in peripheral cells as new potential biomarkers to follow treatment response in Huntington's disease
Source: Clin Transl Med. 2023 Feb 13;13(2):e1154. doi: 10.1002/ctm2.1154 (PMC9925371; doi:10.1002/ctm2.1154)
Supplement: Supplementary file 6 — Supporting Information [file CTM2-13-e1154-s006.docx]

| **ID** | **Gender** | **Age (years)** | **CAG repeats** | **Stage** | **Depression** |
| --- | --- | --- | --- | --- | --- |
| 001 | Female | 43 | - | 0 | 0 |
| 002 | Female | 43 | - | 0 | 0 |
| 003 | Female | 62 | - | 0 | 0 |
| 004 | Female | 41 | - | 0 | 0 |
| 005 | Male | 26 | - | 0 | 0 |
| 006 | Female | 45 | - | 0 | 0 |
| 007 | Female | 49 | 43 | 1 | 0 |
| 008 | Female | 41 | 43 | 1 | 0 |
| 009 | Female | 69 | 42 | 1 | 0 |
| 010 | Female | 56 | 41 | 1 | 0 |
| 011 | Female | 37 | 44 | 1 | 0 |
| 012 | Female | 40 | 42 | 1 | 1 |
| 013 | Female | 69 | 40 | 2 | 0 |
| 014 | Male | 43 | 41 | 2 | 0 |
| 015 | Female | 39 | 45 | 2 | 1 |
| 016 | Male | 43 | 41 | 2 | 1 |
| 017 | Male | 59 | 41 | 2 | 1 |
| 018 | Female | 40 | 49 | 2 | 1 |
| 019 | Male | 59 | 46 | 3 | 1 |
| 020 | Female | 43 | 42 | 3 | 1 |
| 021 | Male | 62 | 41 | 3 | 1 |
| 022 | Female | 70 | 41 | 3 | 1 |

**Table S1 Details on human fibroblast samples used**

Information about the gender, age, CAG repeat length ( -, no information available), stage of disease (0, control; 1, pre-symptomatic; 2, initial; 3, moderated-advanced), and presence of depression (0, no depression; 1, depression)

**Table S2 Details on human PBMCs used**

| **ID** | **Gender** | **Age (years)** | **CAG repeats** | **Stage** | **Depression** |
| --- | --- | --- | --- | --- | --- |
| 201 | Female | 54 | - | 0 | 0 |
| 202 | Female | 25 | - | 0 | 0 |
| 203 | Male | 34 | - | 0 | 0 |
| 204 | Female | 37 | 55 | 3 | 0 |
| 205 | Female | 26 | 64 | 3 | 1 |
| 206 | Male | 82 | 39 | 3 | 1 |
| 207 | Female | 37 | 44 | 1 | 1 |
| 208 | Female | 52 | - | 0 | 0 |
| 209 | Male | 44 | - | 0 | 0 |
| 210 | Male | 66 | 41 | 2 | 0 |
| 211 | Female | 55 | 45 | 3 | 1 |
| 212 | Male | 28 | - | 0 | 0 |
| 213 | Female | 22 | - | 0 | 0 |
| 214 | Female | 45 | - | 0 | 0 |
| 215 | Female | 42 | 45 | 2 | 1 |
| 216 | Male | 60 | 41 | 2 | 0 |
| 217 | Female | 20 | - | 0 | 0 |
| 218 | Male | 53 | - | 0 | 0 |
| 219 | Female | 49 | - | 0 | 0 |
| 220 | Male | 42 | 43 | 2 | 1 |
| 221 | Male | 57 | 43 | 2 | 1 |
| 222 | Male | 29 | 51 | 3 | 1 |
| 223 | Female | 51 | 43 | 2 | 1 |
| 224 | Female | 74 | 40 | 3 | 1 |
| 225 | Female | 70 | 42 | 2 | 0 |
| 226 | Female | 61 | - | 0 | 0 |
| 227 | Female | 47 | 46 | 3 | 1 |
| 228 | Male | 35 | 41 | 2 | 1 |
| 229 | Female | 60 | 41 | 2 | 1 |
| 230 | Male | 74 | 41 | 3 | 1 |
| 231 | Male | 69 | 40 | 2 | 1 |
| 232 | Male | 39 | 44 | 1 | 0 |
| 233 | Female | 40 | 41 | 1 | 1 |
| 234 | Female | 33 | 41 | 1 | 0 |
| 235 | Female | 32 | 43 | 1 | 0 |

Information about the gender, age, CAG repeat length ( -, no information available), and stage of disease (0, control; 1, pre-symptomatic; 2, initial; 3, moderated-advanced).
