# Supplementary material for: Lamin B1 and nuclear morphology in peripheral cells as new potential biomarkers to follow treatment response in Huntington's disease
Source: Clin Transl Med. 2023 Feb 13;13(2):e1154. doi: 10.1002/ctm2.1154 (PMC9925371; doi:10.1002/ctm2.1154)
Supplement: Supplementary file 7 — Supporting Information [file CTM2-13-e1154-s003.docx]

**METHODS AND MATERIALS**

**Human primary fibroblast cultures and** **peripheral blood mononuclear cells (PBMCs) isolation**

Human primary fibroblast cultures and PBMCs were obtained from HD patients at different stages of the disease, and non-related to HD control individuals, selected from Hospital de la Santa Creu i Sant Pau (Barcelona, Spain) from January 2016 to June 2018 (Table S1 and S2). Informed written consent was obtained from each subject before participation in the study, after the nature, purpose and risks of the study were explained. Primary fibroblast cultures were derived from sterile, non-necrotic skin biopsies, that were processed as previously described.^1^ Briefly, they were washed in Dulbecco’s modified Eagle’s medium (DMEM; Gibco, Dublin, IRL), mechanically fragmented in 1-2 mm diameter pieces and grown in T25 culture flasks in DMEM with 25 mM glucose supplemented with 10% v/v fetal bovine serum (FBS; Biological Industries, Cromwell, CT, USA), 1% v/v penicillin/streptomycin (Gibco, Dublin, IRL) and 1% v/v amphotericin B (Sigma-Aldrich, Saint Louis, MO, USA). When a minimum of 50% confluence was achieved, cells were detached from the flask using pre-warmed trypsin (2.5%) and split 1:2 into larger T75 culture flasks for expansion. For further experiments, all fibroblast cell lines were maintained in DMEM with 25 mM glucose supplemented with 10% v/v FBS, 1% v/v penicillin/streptomycin and 1% v/v amphotericin B.

To isolate PBMCs, whole blood (10 ml for each individual stored for a maximum of 4h in EDTA anticoagulant tubes, BD Biosciences, San Jose, CA, USA) was diluted in 10ml phosphate buffered saline (PBS), layered on the top of 10ml Lymphoprep^TM^ (Stemcell Technologies, Vancouver, CA) and centrifuged at 750 xg 20 min at 25ºC. After centrifugation, PBMCs were collected from the interface between Lymphoprep^TM^ and plasma and washed once in PBS (1500 rpm 15min 4ºC). Pellet containing isolated PBMCs was resuspended in 90% FBS + 10% dimethyl sulfoxide (DMSO; Sigma-Aldrich, Saint Louis, MO, USA) and stored at -80ºC until further analysis.

**HD mouse model**

Male R6/1 transgenic mouse, in a B6CBA background, expressing the exon-1 of mHTT with 115 CAG repeats and their wild-type littermate controls were used for this study. Mouse genotyping and CAG repeat length determination were performed as previously described.^2^The animals were housed together in numerical birth order, with access to food and water *ad libitum* in a colony room kept at 19-22ºC and 40-60% humidity, under a 12:12 light/dark cycle. Data were recorded for analysis by microchip mouse number.

**Mouse primary fibroblast cultures**

Mouse primary fibroblast cultures were obtained from a 1-2 mm piece of mouse ear. After the biopsy, the tissue was rinsed with 70% ethanol followed by PBS containing 1% v/v penicillin/streptomycin. Biopsies were then minced under sterile conditions and incubated in 0.5 ml DMEM with 4 mg/ml collagenase 30 min at 37°C and 5% CO_2_.^3^ Fragments and supernatant were incubated in T25 culture flasks and grown in the same culture medium as human primary fibroblasts. When a minimum of 50% confluence was achieved, cells were detached and maintained for further experiments as previously described for human primary fibroblasts.

**Mouse blood films**

Blood was collected from mice by submandibular vein puncture. A small drop of blood was placed on one side of a slide and spread about 3-4 cm in length with a spreader at an angle of 45º from the slide. After letting them dry, blood films were stored at room temperature (r.t.) until analysis.

**In vitro betulinic acid treatment**

Human primary fibroblast cultures obtained from non-affected individuals and HD patients expressing mHTT with ≥ 42 CAG repeats at passage (P) 6-9 were seeded into 6 well-plates and treated with vehicle (DMSO; Sigma) or betulinic acid (20 µg/ml; Sigma) for 48h. Then, cells were harvested and total protein was extracted Western blot analysis as described below.

**In vivo betulinic acid treatment**

Wild-type and R6/1 mice were treated from 8 to 20 weeks of age with vehicle (90% water, 10% Polysorbate 80) or betulinic acid (50 mg/kg; Sigma) administered by oral gavage, 3 days/week. Biopsies were obtained at 8, 12, 16 and 20 weeks of age, 1 hour after the administration of betulinic acid or vehicle.

**Total protein extraction and Western blot analysis**

Mouse primary fibroblasts at P2-3 and human primary fibroblasts at P6-9 were used to obtain protein extracts. Protein extraction and Western blot were performed as previously described.^4^ After incubation with anti-lamin B1 (overnight at 4ºC; 1:1000; Abcam), membranes were washed with Tris-buffered saline (TBS) containing 0.1% Tween 20 (TBS-T), incubated for 1 h at r.t. with the appropriated horseradish peroxidase-conjugated secondary antibody (1:2000; Promega) and washed again with TBS-T. α-Tubulin (1:10,000; T9026, Sigma-Aldrich) was used as a loading control. Immunoreactive bands were visualized using the Western Blotting Luminol Reagent (Santa Cruz Biotechnology) and quantified by a computer-assisted densitometer (Gel-Pro Analyzer, version 4, Media Cybernetics).

**Immunofluorescence**

P2-3 mouse primary fibroblasts and P6-8 human primary fibroblasts were maintained on 12mm round glass coverslips until a minimum of 50% confluence was achieved. Then, cells were washed with PBS and fixed with 4% paraformaldehyde (PFA) in PBS for 10 min at r.t. To block the action of PFA, cells were incubated with 0.2 M glycine for 20 min at r.t. After quenching with 50mM NH_4_Cl for 10 min, cells were permeabilized in blocking buffer containing 1% bovine serum albumin (BSA) + 0.2% gelatin + 0.2% Triton X-100 in PBS at r.t. Blood films were fixed in an acetone: methanol: ethanol (6:2:2) solution for 20 min, washed in PBS + 1% BSA for 5 min, permeabilized in PBS + 1% BSA + 0.1% saponin for 10 min and blocked in PBS + 1% BSA for 1 hour at r.t. Cells were incubated with anti-lamin B1 (1:200, Abcam, Cambridge, UK) for 30 min at r.t. and blood films overnight at 4ºC. Next, cells were washed with PBS and blood films with PBS + 1% BSA and finally incubated with Cy3 AffiniPure F(ab')2 Fragment Goat Anti-Rabbit IgG, F(ab')2 Fragment Specific (1:300 for cells and 1:150 for blood films, Jackson ImmunoResearch, West Grove, PA, USA). Nuclei were stained with DAPI – Fluoromount. Glass coverslips were examined by using the Olympus BX60 (Olympus, Tokyo, Japan) epifluorescence microscope coupled to an Orca-ER cooled CCD camera (Hamamatsu Photonics, Hamamatsu, Japan) at different magnifications (from 10x to 63x). For human and mouse fibroblasts, triplicates and a minimum of 90 fields/cell line were used to manually asses the percentage of cells with nuclear blebs and non-peripheral lamin B1 immunostaining. Confocal images of blood films were taken using a Leica TCS SP5 laser scanning confocal microscope (Leica Microsystems Heidelberg GmbH, Manheim, DE) as stacks differed in 0.29 μm in Z-axis with a HCX PL APO lambda blue 63x numerical aperture objective and standard pinhole (1 Airy disk). A minimum of 10 fields/blood film were analyzed. For image measurements, ImageJ software (NIH, Bethesda, MD, USA) was used combined with different plugins, including the 3D Objects Counter plugin for the quantification of three-dimensional nuclear morphology parameters.

**Fluorescence-activated cellular suspension imaging (FACSI)**

Fluorescence-activated cellular suspension imaging (FACSI) was de result of combining cellular immunostaining with ImageStream imaging flow cytometer technology.^5,6^

*Human PBMCs immunostaining for FACSI*

Immunostaining of isolated PBMCs from HD individuals and non-HD related control individuals was performed using the FIX & PERM Cell Fixation & Cell Permeabilization Kit (ThermoFisher Scientific, Waltham, MA, USA) following the manufacturer’s instructions. First, PBMCs were incubated for 15 min at r.t. in buffer (PBS + 5% FBS + 0.1% sodium azide) containing the conjugated antibodies directed to the cell surface markers of interest (CD3 Monoclonal Antibody (MEM-57), APC, 1:1000, ThermoFisher Scientific (Waltham, MA, USA); CD14 Monoclonal Antibody (TuK4), Pacific Orange, 1:25, Invitrogen (Carlsbad, CA, USA); CD19 Monoclonal Antibody (SJ25-C1), PE, 1:50, Invitrogen (Carlsbad, CA, USA). Then, 100 μl of Reagent A (Fixation Medium) was added and incubated for 15 min at r.t. After washing in buffer and centrifuge at 1500 rpms for 10 min at 4ºC, cells were incubated with rabbit anti-lamin B1 (1:1000, Abcam, Cambridge, UK) in 100 μl of reagent B for 20 min at r.t. Then, cells were washed and incubated with Alexa Fluor 488 AffiniPure Donkey Anti-Rabbit IgG (H+L) for 15 min at r.t. Finally, cells were washed once in buffer and directly processed for imaging flow cytometry.

*Imaging flow cytometry (Imagestream)*


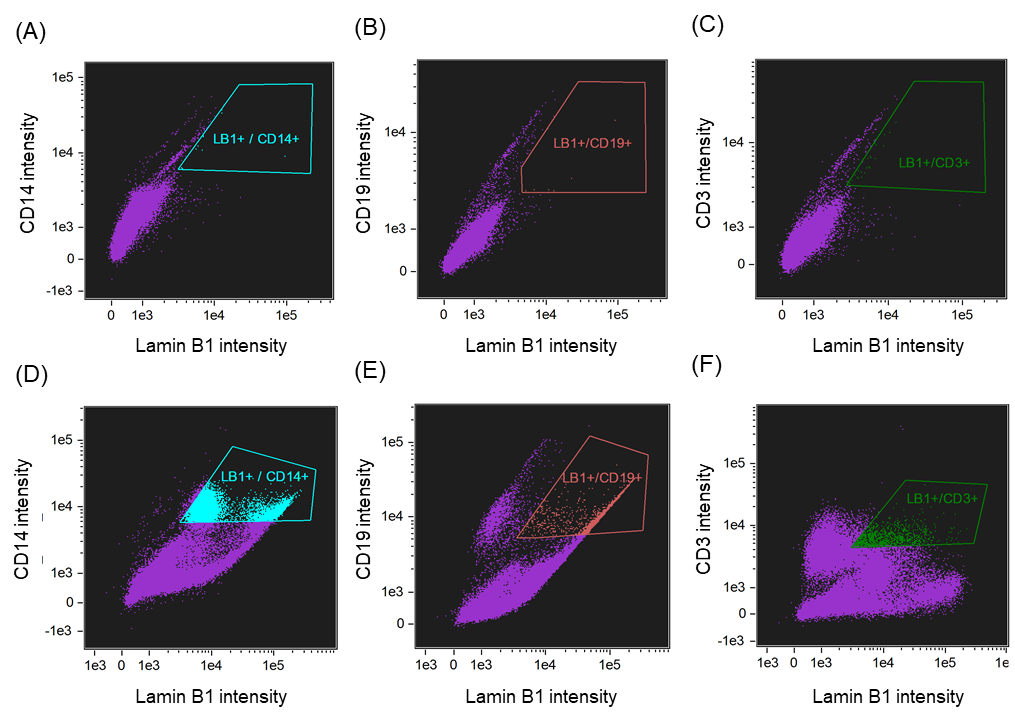
The immunostained PBMCs (from now on, referred to as events) were resuspended in 100 µl and filtered using cells strainers of 100 µm pore size (Sysmex Partec, Kobe, Japan) to remove aggregates and big remnants that could obstruct the cytometer. The samples were then sorted and imaged using a 40x objective at a maximum speed of 600 events/s depending on the sample concentration and a minimum of 10000 events were recorded for each replicate. Different controls were performed to evaluate the specificity of the signal obtained, and of the populations selected by removing one of the antibodies and using the rest (fluorescent minus one, FMO), and as observed in Figure, the signal of the populations depending on the specific antibody removed completely disappeared.


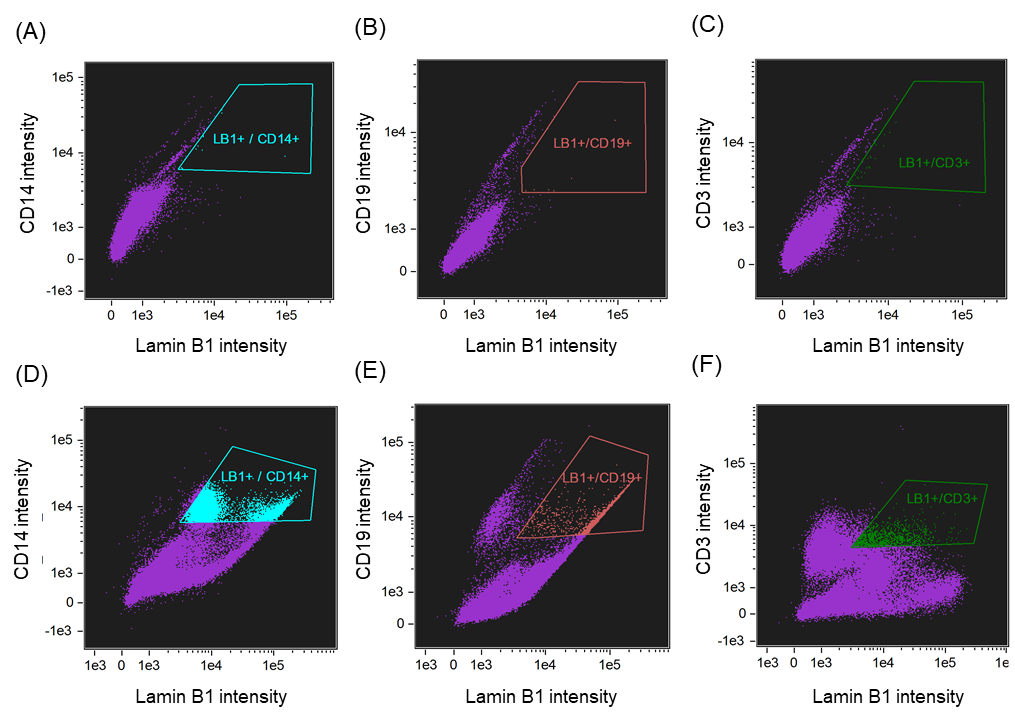


Selection of different populations. Identification of different populations of PBMCs using FMO for the different antibodies (A, CD14; B, CD19; C, CD3). The final populations analyzed are represented (D-F).

The recorded files were processed and analyzed by the IDEAS Software provided by the Imagestream machine’s manufacturers after compensating the unspecific signal for each channel coming from the rest of the channels. From all the focused population, only the events positive for lamin B1 staining were selected for the posterior analysis as described previously.^7^ These lamin B1 positive events were then screened according to their signal levels of different markers and classified into different populations: monocytes (CD14+); B cells (CD19+) and T Cells (CD3+). All the features analyzed later on were performed using these selected populations, including intensity, circularity, and mean area.

**Statistical analysis**

Data were scored and analyzed blinded to genotype. All the results are expressed as the mean ± SEM. Shapiro-Wilk test was used to assess normality. For normally distributed data, Student’s *t* test (equal variance) or Welch’s *t* test (unequal variance) were used. Wilcoxon signed-rank test was used for not normally distributed data. Two-way ANOVA was used for multi-component variables, followed by Bonferroni’s post hoc test as indicated in the figure legends. A 95 % confidence interval was used and values with a p < 0.05 were considered statistically significant.

**REFERENCES**

1. Garbett KA, Vereczkei A, Kálmán S, et al. Coordinated messenger RNA/microRNA changes in fibroblasts of patients with major depression. *Biol Psychiatry*. 2015;77(3):256-265.

2. Mangiarini L, Sathasivam K, Seller M, et al. Exon 1 of the HD gene with an expanded CAG repeat is sufficient to cause a progressive neurological phenotype in transgenic mice. *Cell*. 1996;87(3):493-506.

3. Khan M, Gasser S. Generating Primary Fibroblast Cultures from Mouse Ear and Tail Tissues. *J Vis Exp*. 2016;2016(107).

4. Saavedra A, Fernández-García S, Cases S, et al. Chelerythrine promotes Ca 2+-dependent calpain activation in neuronal cells in a PKC-independent manner. *Biochim Biophys Acta Gen Subj*. 2017;1861(4):922-935.

5. Benito E, Urbanke H, Ramachandran B, et al. HDAC inhibitor-dependent transcriptome and memory reinstatement in cognitive decline models. *J Clin Invest*. 2015;125(9):3572-3584.

6. Vorobjev IA, Barteneva NS. Quantitative Functional Morphology by Imaging Flow Cytometry. *Methods Mol Biol*. 2016;1389:3-11.

7. Alcalá‐Vida R, Garcia‐Forn M, Castany‐Pladevall C, et al. Neuron type-specific increase in lamin B1 contributes to nuclear dysfunction in Huntington’s disease. *EMBO Mol Med*. 2021;13(2)
